# Exergaming Improves Executive Functions in Patients With Metabolic Syndrome: Randomized Controlled Trial

**DOI:** 10.2196/13575

**Published:** 2019-07-31

**Authors:** Shanshan Wu, Eun-Ah Jo, Hongqing Ji, Kyung-Hee Kim, Jung-Jun Park, Bo Hyun Kim, Kyoung Im Cho

**Affiliations:** 1 Division of Sport Science Pusan National University Busan Republic of Korea; 2 Department of Internal Medicine Kosin University College of Medicine Busan Republic of Korea; 3 Department of Cardiology Sejong General Hospital Bucheon Republic of Korea; 4 Department of Internal Medicine Pusan National University Hospital and Biomedical Research Institute Busan Republic of Korea; 5 Division of Cardiology Department of Internal Medicine Kosin University College of Medicine Busan Republic of Korea

**Keywords:** exercise, executive functions, event-related potential, games, metabolic syndrome

## Abstract

**Background:**

Recent studies indicate that participation in exercise-related games can improve executive function, attention processing, and visuospatial skills.

**Objective:**

The aim of this study was to investigate whether exercise via exergaming (EXG) can improve executive function in patients with metabolic syndrome (MetS).

**Methods:**

A total of 22 MetS patients were recruited and randomly assigned to an EXG group or a treadmill exercise (TE) group. The reaction time (RT) and electrophysiological signals from the frontal (Fz), central (Cz), and parietal (Pz) cortices were collected during a Stroop task after 12 weeks of exercise.

**Results:**

During the Stroop congruence (facilitation) judgment task, both the EXG and TE groups showed significantly faster RT after 12 weeks of exercise training. For N200 amplitude, the EXG group demonstrated significantly increased electrophysiological signals from the Fz and Cz cortices. These changes were significantly larger in the EXG group than in the TE group. Separately, for the P300 amplitude, the EXG groups presented significantly increased electrophysiological signals from the Fz, Cz, and Pz cortices, whereas the TE group showed significantly increased electrophysiological signals from the Cz and Pz cortices only. During the Stroop incongruence (interference) judgment task, both the EXG and TE groups showed significantly faster RT. For P300 amplitude, the EXG group had significantly increased electrophysiological signals from the Fz and Cz cortices only, whereas the TE group had significantly increased electrophysiological signals from the Fz, Cz, and Pz cortices.

**Conclusions:**

EXG improves executive function in patients with MetS as much as normal aerobic exercise does. In particular, a unique benefit of EXG beyond increased aerobic capacity is the improved selective attention among cognitive functions. Thus, EXG could be recommended to someone who needs to improve their brain responses of concentration and judgment as well as physical fitness.

**Trial Registration:**

ClinicalTrials.gov NCT04015583; https://clinicaltrials.gov/ct2/show/NCT04015583

## Introduction

### Background

In recent years, the relationship between cognitive function and metabolic syndrome (MetS) has been widely studied [1,2]. MetS has been found to be associated with a decline in areas related to executive function [3,4] because of multiple risk factors, including hypertension, dyslipidemia, impaired glucose homeostasis, and abdominal obesity. Executive functions include basic cognitive processes such as attentional control, cognitive inhibition, inhibitory control, working memory, and cognitive flexibility [5].

Research on cognitive neuroscience employs Stroop tasks to measure selective attention capacity and skills as well as process speed ability to elucidate the nature of executive functions [6]. Electroencephalographic (EEG) activity using event-related positioning technology has been widely used to measure selective attention capacity and skills, and evaluations of behavioral performance such as reaction time (RT) are commonly used to determine processing speed ability [7,8]. N200 negativity (200-350  milliseconds [ms] poststimulus) is an event-related potential (ERP) indicating the attentional capacity that is usually induced before motion response control and is related to the cognitive processes of stimulus recognition and differentiation [9]. P300 positivity (300-600 ms poststimulus) is another ERP that reflects memory-related neural processing and is involved in categorizing incoming information and updating the context of the working memory (eg, encoding, rehearsal, recognition, and retrieval) [10].

It is well-known that aerobic exercise training provides various beneficial clinical outcomes in metabolic disease patients [11,12]. Its effects on cognitive function, especially executive function, have also been investigated [13]. Furthermore, recent studies reported that both aerobic and resistance exercise training facilitate overall electrophysiological effects (eg, increased P300 amplitudes) and behavior changes (eg, faster RT) in otherwise healthy elderly people [14,15]. In addition, aerobic exercise has been suggested to improve cognitive processes in cortical cognitive control (P300 amplitude) in studies involving chronic stroke patients [16].

### Exergaming

Recently, exergaming (EXG, a combination of *exercise* and *gaming*) has attracted much attention as a novel exercise method in terms of improving cognitive function because it utilizes video games that require body movements while simultaneously presenting the user with a cognitively challenging environment [17]. Along with its popular usage for leisure and entertainment, there is a growing interest in the application of EXG to improve clinical outcomes. Recent studies using EXG showed its beneficial effects on cognitive and dual-task functions, which reduced falls in older adults [18] as well as cardiovascular disease risks, such as body fat, serum adipokine levels, and lipid profiles [19]. EXG also promoted improved executive functions and cognitive processing speed in both elderly people and children [20,21]. This growing evidence suggests that EXG offers the benefit of improving both cognitive and physical functions.

Although many previous studies have reported improvements in cognitive function following EXG, it is not clear whether this benefit is due to an exercise effect or video game effect. In addition, all of these studies measured RT instead of ERP using EEG, which limits investigators seeking to illuminate brain activities. Considering that EEG can measure electrical activities in various cortex areas in the brain, it is necessary to investigate ERP using EEG to evaluate executive function. Therefore, we examined the benefits of EXG in comparison with normal exercise and investigated executive function by measuring RT as well as N200 and P300 in 3 cortex areas via Stroop tasks applied in patients with MetS.

## Methods

### Participants

A total of 22 MetS male and female patients aged between 50 and 80 years participated in this study. MetS was defined according to the modified National Cholesterol Education Program Adult Treatment Panel III definition for South Asians. Briefly, individuals with 3 or more of the following criteria were defined as having MetS: central obesity (waist circumference ≥90 cm for men or ≥85 cm for women); fasting plasma glucose ≥100 mg/dL or current treatment for diabetes mellitus; systolic blood pressure ≥130 mmHg, diastolic blood pressure ≥85 mmHg, or current treatment for hypertension; serum triglyceride level ≥150 mg/dL; and low high-density lipoprotein cholesterol (<40 mg/dL for men or <50 mg/dL for women) [22]. Subjects were asked not to exercise for 24 hours before the experiment. They were also instructed to eat usual meals and to finish meals at least four hours before the experiment, while avoiding alcohol for a day before the experiment and caffeine during the 4 hours before the experiment. All subjects were required to complete a written informed consent form approved by the Institutional Review Board of Kosin University College of Medicine.

The sample size was calculated using a sample size calculation software program (G*Power version 3.1.9.2 for Windows), with an effect size of 0.484, statistical power of .80, and statistical level of significance of .05. The effect size was calculated from previous studies [13,14]. As a result, the sample size for each group was established at 8 patients, so we decided to recruit 11 patients for each group in consideration of a potential 30% (3/11) dropout rate.

### Exercise Training Interventions

Exercise training was conducted at Kosin University Gospel Hospital. Each participant was instructed to immediately inform the study supervisor if he or she experienced any unusual symptoms during exercise training and to consult a physician if needed. Subjects were excluded from the final analysis if they did not perform more than 80% of the exercise sessions.

All subjects were randomly stratified into either an EXG group or a treadmill exercise (TE) group. Subjects underwent 2 weeks of adaptation and then carried out 12 weeks of exercise training for 60 min per day, 3 days per week, at 60% to 80% of their heart rate reserve (HRR). Each exercise session consisted of 10 min of warm-up, 40 min of main exercise, and 10 min of cooldown.

**Figure 1:**
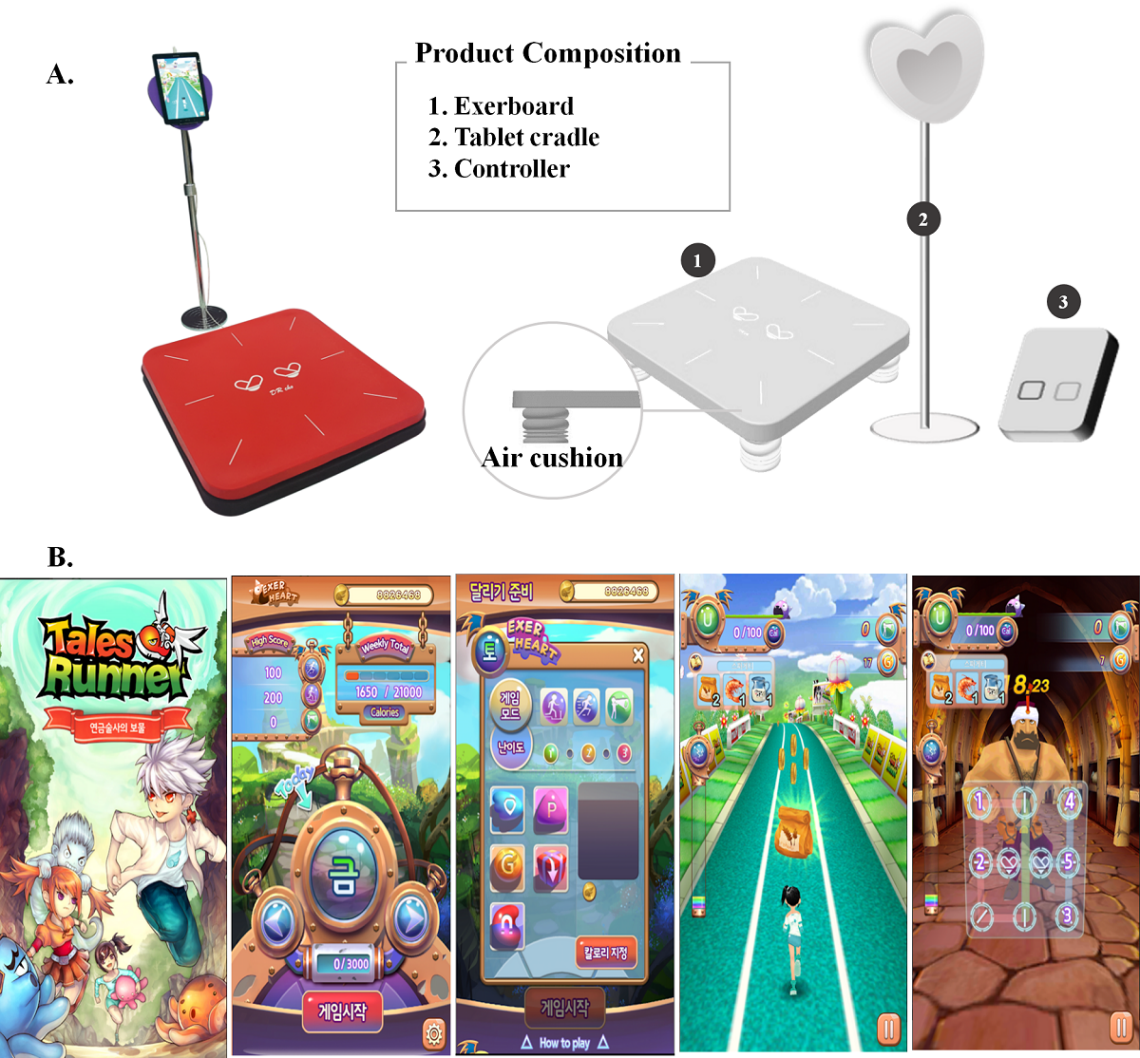
(A) The exergaming group performed exercise using Exerheart devices with permission from D&J Humancare, who is the copyright holder of Exerheart. (B) Features of the video game "Alchemist's Treasure.".

The EXG group performed exercise using the Exerheart equipment (D&J Humancare) that is composed of a running and jumping mat (730 width × 730 depth × 130 height) and a tablet personal computer placed on a stand (which could be adjusted to any height between 70 and 155 cm; Figure 1). Exerheart is an EXG system developed for in situ running along with the video game called *Alchemist's Treasure* (D&J Humancare; Figure 1). To play this game, the subject has to run or jump on a spot on the mat to move a virtual avatar on the screen of the tablet computer to the front, back, left, and right along with music (Multimedia Appendix 1). The subject can control the speed of their avatar’s movement via their running or jumping speed on the mat. Participants in the TE group performed exercise using commercial treadmills (MOTUS). Each subject walked or ran on their treadmill at a comfortable speed.

For both the EXG and TE groups, all subjects’ heart rates (HRs) during exercise were monitored using HR monitors (Polar RS400sd) to confirm that the value was within the target HR range. The Karvonen formula [23] was used to calculate the HRR (estimated maximal HR−resting HR) and the target HR during exercise (HRR × given percentage of training intensity + resting HR).

### Stroop Test

To assess executive function, a computer-based version of the Stroop task was administered using the Telescan software (LAXTHA Inc). During the task, subjects were presented with a color word appearing in the same color on congruent trials (eg, *blue* printed in blue) and in a different color on incongruent trials (eg, *blue* printed in green) [24]. To provide similar visual content, blue, green, and yellow colors were chosen as stimuli.

Subjects performed the Stroop task twice, before and after exercise training. Subjects sat 1 m from the screen and when the color words appeared on the screen, they clicked the left keyboard for the congruent test and the right keyboard for the incongruent test. Subjects were instructed to respond as quickly and accurately as possible. The rate of measurement targeted for 50%. Each color word (vertical viewing angle: 2 degrees) was presented for 200 ms and a response was accepted within 1500 ms. The interstimulus interval varied randomly between 1500 and 2500 ms.

### Electroencephalographic Measurements

EEG activity was recorded during the modified Stroop task by using a computerized polygraph system (type A: a total of 31 channels Poly G-As, LAXTHA). Silver chloride electrodes (LAXTHA) were placed on the frontal (Fz), central (Cz), and parietal (Pz) cortex areas, according to the international 10-20 system. Midline locations were referenced to link earlobe electrodes. Horizontal and vertical electrooculograms were monitored by electrodes placed above and below the left eye and at the outer canthus of both eyes, respectively. The impedance of all electrodes was maintained below 10 kΩ. The bandpass filter of the amplifier was 0.1 to 100 Hz, the sampling rate was 1000 Hz, and a notch filter was established at 60 Hz.

The N200 component was defined as the largest positive peak occurring between 200 and 350 ms poststimulus, whereas the P300 component was defined as the largest positive peak occurring between 300 and 600 ms poststimulus [7]. N200 and P300 amplitudes were measured as the differences between the mean prestimulus baseline and maximum peak amplitude. Telescan’s built-in high-pass infinite impulse response filter was used for filtering. Waveforms were digitally smoothed with a low-pass filter using a half power cutoff of 10 Hz before analysis.

### Statistical Analysis

Owing to the small sample size of this study, we used nonparametric statistics for data analysis. We used the Wilcoxon signed rank test to examine the changes of each dependent variable after the intervention within each group. The Mann-Whitney U test was employed to compare the delta values between training groups (Δ‐EXG group vs Δ‐TE group). The effect size of partial eta squared (η2) was reported for significant effects, where the alpha level for all of the tests was set at .05. Data were expressed as mean (SD). All statistical tests were processed using the Statistical Package for the Social Sciences version 24 software program (IBM Corp).

## Results

Demographic and physical characteristics for all subjects are provided in Table 1. There were no significant group differences noted during baseline measurements.

### Reaction Time

**Figure 2:**
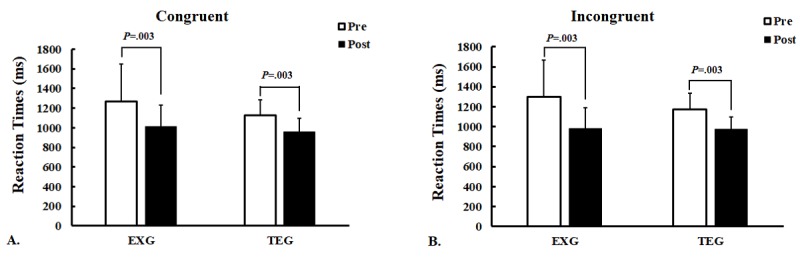
Mean reaction time during the Stroop task in the exergaming and treadmill exercise groups before and after the exercise intervention. (A) Congruent; (B) incongruent. EXG: exergaming; TEG: treadmill exercise group; ms: milliseconds. **Significant difference at P<.01 (Wilcoxon signed-rank test).

The changes in congruent RT after 12 weeks of exercise training were not significantly different between the EXG and TE groups (Table 2): both showed significantly shortened congruent RT (Figure 2). Similarly, the changes in incongruent RT after 12 weeks of exercise training were not significantly different between the EXG and TE groups, in that both showed significantly shortened incongruent RT (Figure 2).

**Table 1:** Baseline characteristics of study participants.

Factor	Group		*P* value
	Exergaming (n=11), mean (SD)	Treadmill exercise (n=11), mean (SD)	
Age (years)	64 (10)	60 (7)	.30
Height (cm)	154.24 (5.73)	161.66 (7.1)	.01
Weight (kg)	69.38 (10.54)	71.47 (11.62)	.66
Body mass index (kg/m^2^)	29.07 (3.3)	27.31 (3.52)	.24
Waist circumference (cm)	97.36 (10.95)	93.6 (8.78)	.40
Glucose (mg/dl)	123.55 (25.68)	112.36 (29.37)	.35
High-density lipoprotein cholesterol (mmol/L)	46.32 (8.91)	50.59 (10.52)	.32
Low‐density lipoprotein cholesterol (mmol/L)	66.19 (22.8)	78.38 (19.52)	.19
Total cholesterol (mmol/L)	134.81 (25.52)	146.67 (12.04)	.18
Triglycerides (mmol/L)	136.82 (78.88)	145.73 (142.74)	.86
Systolic blood pressure (mmHg)	127.09 (16.86)	128.09 (17.39)	.89
Diastolic blood pressure (mmHg)	75.55 (9.43)	77.73 (11.99)	.64

**Table 2:** Comparison of Stroop task congruent and incongruent reaction times of the exergame group and treadmill exercise group.

Effects, group		Pre, mean (SD)	Post, mean (SD)	Wilcoxon signed rank test^a^, *P* value	Chi-square value	Mann-Whitney U test^b^, *P* value
**Congruent**						**.28**
	EXG^c^ group	1265.91 (383.15)	1012.09 (221.64)	.01	0.053	—^d^
	TE^e^ group	1124.18 (161.21)	957.82 (138.05)	.01	—	—
**Incongruent**						**.67**
	EXG group	1299.09 (367.48)	984.73 (204.81)	.01	0.008	—
	TE group	1171.36 (163.26)	974.55 (120.97)	.01	—	—

^a^Comparison of pre versus post within group.

^b^Comparison of the delta values between EXG and TE group.

^c^EXG: exergaming.

^d^Not applicable.

^e^TE: treadmill exercise.

### Event-Related Potential Data

#### N200 Amplitude

**Figure 3:**
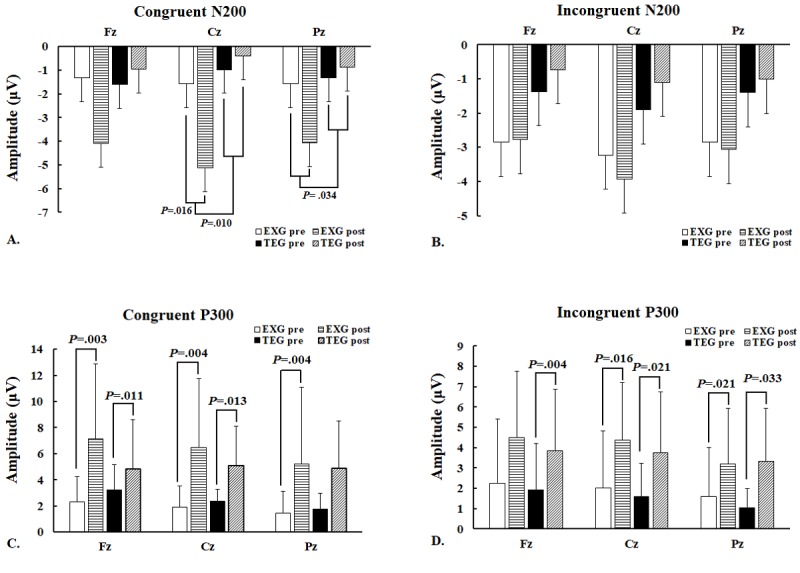
N200 and P300 amplitudes (mean [SE]) on 3 electrodes during the Stroop task in the exergaming and treadmill exercise groups before and after 12 weeks of exercise training. (A) Congruent N200 amplitudes; (B) incongruent N200 amplitudes; (C) congruent P300 amplitudes; (D) incongruent P300 amplitudes. Fz: frontal cortex; Cz: central cortex; Pz: parietal cortex; EXG: exergaming group; TEG: treadmill exercise group.

According to the results in Table 3, after 12 weeks of exercise training, the increases in congruent N200 amplitude on Cz and Pz cortices in the EXG group were significantly greater than in the TE group, but this was not true with regard to Fz. In addition, EXG significantly increased congruent N200 amplitude on Cz, but not for Fz and Pz. On the contrary, the TE group showed no significant changes in congruent N200 amplitude for Fz, Cz, or Pz (Figure 3).

The changes in incongruent N200 amplitude for Fz, Cz, and Pz after 12 weeks of exercise training were not significantly different between the EXG and TE groups (Table 3). Interestingly, EXG did not significantly change the incongruent N200 amplitude for Fz, Cz, or Pz, whereas TE also did not significantly change the incongruent N200 amplitude for Fz, Cz, or Pz (Figure 3). The waveforms of congruent and incongruent N200 amplitudes for Fz, Cz, and Pz for the EXG and TE groups before and after exercise are shown in Figure 3.

**Table 3:** Comparison of Stroop task congruent and incongruent N200/P300 amplitudes of the exergame group and treadmill exercise group.

Components, effects, and group				Pre, mean (SD)	Post, mean (SD)	Wilcoxon signed rank test^a^, *P* value	*η2*^b^	Mann-Whitney U test^c^, *P* value
**N200 amplitude**								
	**Congruent**							
		**Fz^d^**						
			EXG^e^ group	−1.3 (1.95)	−4.1 (3.02)	.09	0.138	.09
			TE^f^ group	−0.98 (3.14)	−0.41 (3.82)	.33	—^g^	—
		**Cz^h^**						
			EXG group	−1.59 (2.54)	−5.13 (2.94)	.03	0.291	.01
			TE group	−1.61 (3.89)	−0.97 (4.59)	.42	—	—
		**Pz^i^**						
			EXG group	−1.58 (1.89)	−4.06 (2.89)	.06	0.207	.03
			TE group	−1.32 (3.62)	−0.89 (3.99)	.53	—	—
	**Incongruent**							
		**Fz**						
			EXG group	−2.85 (2.3)	−2.78 (3.02)	.48	0.041	.37
			TE group	−1.37 (3.3)	−0.73 (3.18)	.42	—	—
		**Cz**						
			EXG group	−3.23 (2.69)	−3.93 (2.92)	.29	0.099	.15
			TE group	−1.9 (3.82)	−1.1 (3.65)	.21	—	—
		**Pz**						
			EXG group	−2.86 (2.45)	−3.07 (2.62)	.72	0.008	.70
			TE group	−1.4 (3.66)	−1.01 (3.52)	.48	—	—
**P300 amplitude**								
	**Congruent**							
		**Fz**						
			EXG group	2.3 (1.94)	7.12 (5.73)	.01	0.008	.70
			TE group	3.21 (1.95)	4.82 (3.78)	.01	—	—
		**Cz**						
			EXG group	1.92 (1.63)	6.49 (5.28)	.01	0.006	.75
			TE group	2.36 (0.93)	5.08 (3.03)	.01	—	—
		**Pz**						
			EXG group	1.44 (1.69)	5.2 (5.88)	.01	0.099	.15
			TE group	1.74 (1.26)	4.87 (3.64)	.33	—	—
	**Incongruent**							
		**Fz**						
			EXG group	2.26 (3.14)	4.48 (3.27)	.09	0.018	.56
			TE group	1.93 (2.26)	3.85 (3.04)	.01	—	—
		**Cz**						
			EXG group	2.03 (2.8)	4.38 (2.81)	.02	0.002	.85
			TE group	1.59 (1.63)	3.74 (2.99)	.02	—	—
		**Pz**						
			EXG group	1.58 (2.44)	3.2 (2.73)	.02	0.000	.95
			TE group	1.05 (0.92)	3.33 (2.59)	.03	—	—

^a^Comparison of pre versus post within group.

^b^*η2*: chi-square test value.

^c^Comparison of the delta values between EXG and TE group.

^d^Fz: frontal cortex.

^e^EXG: exergame.

^f^TE: treadmill exercise.

^g^Not applicable.

^h^Cz: central cortex.

^i^Pz: parietal cortex.

#### P300 Amplitude

Table 3 shows that the results of the Mann-Whitney U test for changes in the congruent P300 amplitude for Fz, Cz, and Pz after 12 weeks of exercise training were not significantly different between the EXG and TE groups. EXG significantly increased the congruent P300 amplitude for Fz, Cz, and Pz. However, TE significantly increased the congruent P300 amplitude only for Fz and Cz (Figure 3).

**Figure 4:**
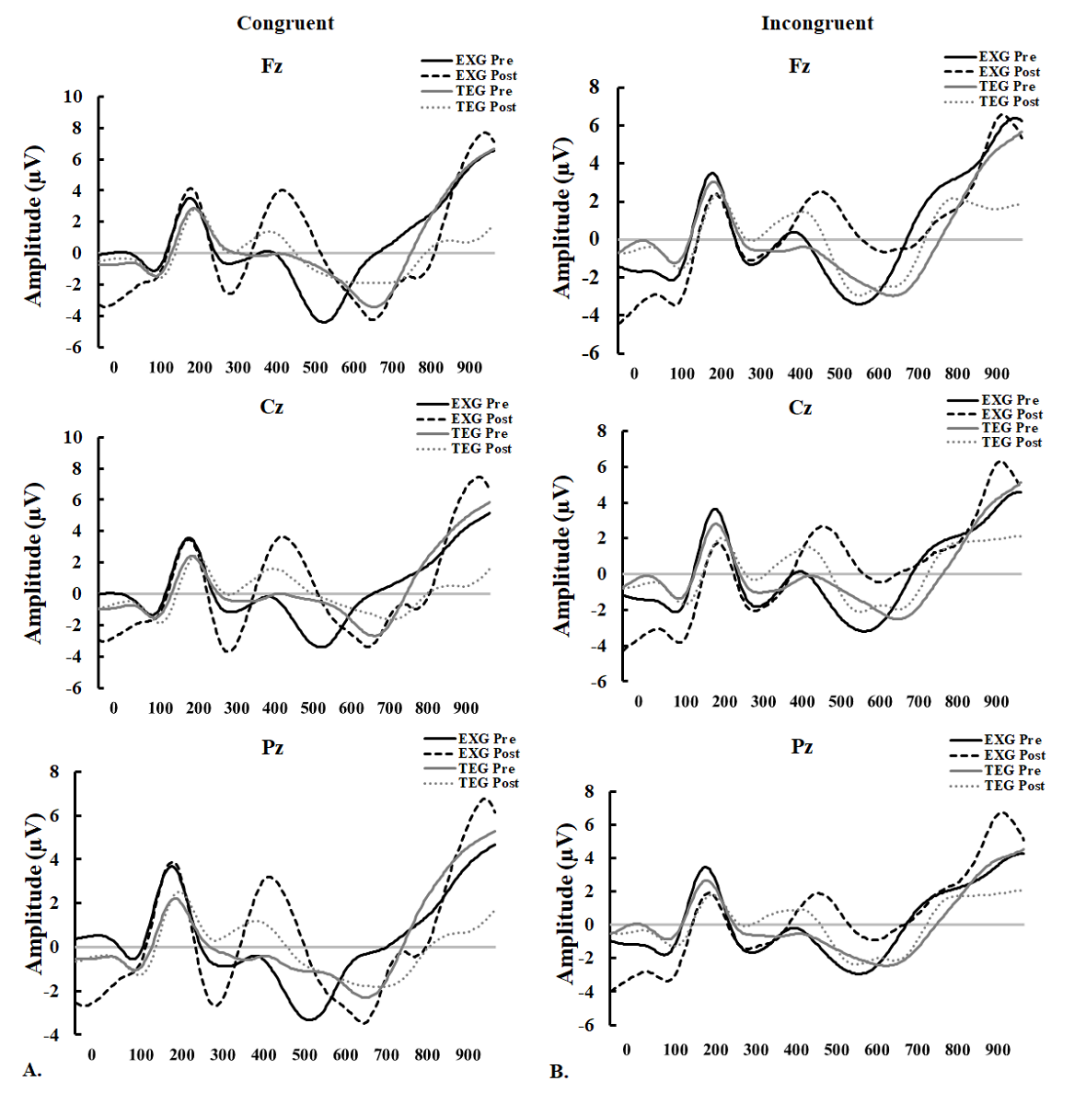
Average event-related potential waveforms of electrodes for mean N200 and P300 amplitudes during the Stroop task in the exergaming and treadmill exercise groups before and after 12 weeks of the exercise training. (A) Congruent; (B) incongruent. Fz: frontal cortex; Cz: central cortex; Pz: parietal cortex; EXG: exergaming group; TEG: treadmill exercise group; ms: milliseconds.

There were no significant differences in the changes in the incongruent P300 amplitude for Fz, Cz, and Pz between the EXG and TE groups after 12 weeks of exercise training (Table 3). EXG significantly increased the incongruent P300 amplitude for Cz and Pz, but not for Fz. On the contrary, TE significantly increased the incongruent P300 amplitude for Fz, Cz, and Pz (Figure 3). The waveforms of congruent and incongruent P300 amplitudes for Fz, Cz, and Pz for the EXG and TE groups before and after exercise are shown in Figure 4.

## Discussion

### Principal Findings

This study was the first to investigate the benefits of EXG in comparison with normal exercise on the behavioral performance and executive function of patients with MetS. We found that 12 weeks of both EXG and TE training similarly and effectively improved behavioral performance and congruent and incongruent memory-related neural processing. However, only EXG training improved congruent selective attention, whereas neither EXG nor TE training affected incongruent selective attention. These results suggest similar overall effects for EXG and normal exercise on behavioral performance and executive function in patients with MetS but that EXG could be more effective than normal exercise for congruent selective attention.

This study showed that both 12 weeks of EXG and TE training effectively improved RT in MetS patients, and these changes were not different between the 2 training protocols. These results suggest that both EXG and normal exercise are able to improve behavioral performance, but EXG does not have more beneficial effects compared with normal exercise. In our previous study, we examined the performance of control tasks using a simple acute aerobic exercise and complex exercise [25]. The results indicated that participants did not have a difference in performance when participating in the simple exercise compared with very skilled complex exercises. In another study, contrary to our results, an acute single-bout study comparing the effects of standard normal exercise with EXG on attention performance in young adults found no significant improvement with EXG [7]. The results of this previous study suggest that, after 20 min of unskilled WiiFit training, the brain requires further information processing capabilities, which may be the source of control requirements and pressure increases, offsetting the potential benefits of the exercise component. However, compared with the WiiFit system, EXG with Exerheart involves running-based aerobic exercise on an air cushion board with game-based contents, such as adventures, racing, and quizzes, which continue to arouse the user’s interest in exercise. So, with Exerheart, an individual is running constantly with changing visual stimuli; these repeated effects simultaneously increase physical activity and cognitive function via an interactive virtual reality engagement [20]. Therefore, through EXG and TE, the RT of Stroop task conditions of MetS patients could be shortened, which would promote basic information processing and the executive function of suppression control.

In this study, the congruent and incongruent P300 amplitudes were increased after 12 weeks of both EXG and TE training, with no difference seen between the 2 groups. These results indicate that both EXG and normal exercise improve memory-related neural processing, but the beneficial effects of EXG are more significant than normal exercise. In other words, participation in exercise, regardless of the exercise modality, induces an increase in working memory in executive function. However, our previous study showed that P300 amplitude increased during a control task following futsal relative to seated rest or TE, indicating that complex control of the brain stimulates the executive control network of the cortex [25]. It was found that as one’s age increases, the P300 amplitude in the central (Cz) region decreases and the scalp distribution of the P300 amplitude is transferred to the frontal region [26]. Pontifex et al [27] examined P300 components and found that older adults with high cardiorespiratory fitness only exhibited greater P300 amplitudes, whereas Tsai et al [28] revealed that different exercise types have greater P300 amplitudes for older individuals. However, as they pointed out, regardless of the type of older people participating in sports, physical exercise is a lifestyle factor that is crucial to preventing age-related biological degeneration in the frontal-to-parietal areas, thus delaying the cognitive declines associated with later life. Considering that EXG is a kind of aerobic exercise, it stands to reason that EXG could improve not only cardiovascular health but also cognitive plasticity, thereby improving categorization of the incoming information and updating the context of working memory in MetS patients.

We found that neither EXG nor TE training affected the incongruent N200 amplitude. However, the consistent N200 amplitude was increased by EXG training. These findings suggest that, although neither EXG nor normal exercise affected incongruent selective attention, EXG improved congruent selective attention, which suggested that EXG has a more beneficial effect on congruent selective attention compared with normal exercise. The results of many studies on the relationship between exercise and the N200 amplitude indicate that exercise has no significant effect on the N200 amplitude [28,29]. Pontifex et al showed that general decreases in N200 amplitudes across scalp sites were observed during exercise relative to rest [29]. The N200 component plays a key role in the anterior cingulate cortex (ACC), which is part of the potential prefrontal cortex and regulates dopaminergic neurons in cognitive functions, such as working memory, attention, and decision making [30-32]. Therefore, the reduction of N200 amplitude caused by normal aerobic exercise severely limits ACC activity [29]. In light of our N200 amplitude findings, these results suggest that EXG better regulates the activity of ACC in the prefrontal cortex than does aerobic exercise, thereby effectively increasing consistent selective attention.

Recent studies suggest that combining motor and cognitive demands during exercising can improve cognitive function more so than training these domains separately [17,33]. In addition, cognitive video game training can have beneficial effects on memory, attention, and RT in older adults [34,35]. In previous studies, when participants consistently performed exercises in a virtual environment, an increase in the N200 amplitude positively promoted decision making (frontal and central) and visual perception (occipital) [36]. Therefore, EXG positively promotes visual perceptual stimulation in the virtual environment to enhance the selective attention activity associated with the cerebral cortex, thereby strongly promoting executive function. Exercise and video games can each improve brain structure and function [37-40]; thus, their combination can have a complementary effect on brain stimulation and protection.

Our study provides evidence that EXG improves RT and incongruent memory-related neural processing in MetS patients as much as normal aerobic exercise does. In addition, EXG improves congruent selective attention, which was not changed by normal aerobic exercise. Therefore, EXG could provide an innovative way to enjoy aerobic exercise compared with repetitive, conventional exercises.

### Limitations

Although this study found significant results, there were some limitations: (1) The sample size in this study was relatively small (2) The age range was relatively large at 50 to 80 years. Considering that, with age, response time and brain activity become slower, we cannot rule out the possibility that age will affect the performance of executive function. However, the mean age was similar in both groups, so this possibility might be low in this study; (3) Finally, the intensity of Exerheart use while playing the *Alchemist's Treasure* game was unable to be controlled in a standard fashion.

### Conclusions

The results of this study suggest that EXG enhances brain responses to concentration and judgment, resulting in increased behavioral response among MetS patients comparable with the impact of normal aerobic exercise. Furthermore, the unique advantage of EXG is that it improves selective attention among cognitive functions, unlike normal aerobic exercise. Therefore, EXG could be recommended to some patients who need to improve executive function as well as physical fitness.

## Appendix

Multimedia Appendix 1 Participants played the video game with Exerheart.

Multimedia Appendix 2 CONSORT-eHEALTH checklist (V 1.6.1).

## Abbreviations

ACC: anterior cingulate cortex
Cz: central cortex
EEG: electroencephalographic
ERP: event-related potential
EXG: exergaming
Fz: frontal cortex
HR: heart rate
HRR: heart rate reserve
MetS: metabolic syndrome
ms: milliseconds.
Pz: parietal cortex
RT: reaction time
TE: treadmill exercise
TEG: treadmill exercise group
